# A scoping review of socio-cultural factors affecting tuberculosis control: evidence from global studies

**DOI:** 10.3389/fpubh.2025.1704805

**Published:** 2026-01-08

**Authors:** Amidu Alhassan, Yula Salifu, Patience Fakornam Doe, Frank Offei Odonkor, Joseph Lasong

**Affiliations:** 1 Department of Adult Health, School of Nursing and Midwifery, College of Health and Allied Sciences, University of Cape Coast, Cape Coast, Ghana; 2 Department of Nursing, School of Biomedical Engineering and Allied Health Sciences, All Nations University, Koforidua, Ghana; 3 Department of Population, Family and Reproductive Health, School of Public Health, University of Ghana, Accra, Ghana; 4 Department of Public Health, School of Nursing and Midwifery, College of Health and Allied Sciences, University of Cape Coast, Cape Coast, Ghana; 5 Department of Population and Reproductive Health, School of Public Health, University for Development Studies, Tamale, Ghana

**Keywords:** tuberculosis, socio-cultural factors, health-seeking behaviour, TB control, socioeconomic determinants

## Abstract

**Background:**

Tuberculosis (TB) remains a pressing global health challenge, ranking among the leading causes of death from a single infectious agent. In 2021, the World Health Organisation (WHO) estimated 10.6 million new TB cases and 1.6 million TB-associated deaths globally, underscoring the enduring burden of the disease. The review aimed to explore and map the socio-cultural factors affecting TB control by synthesising evidence from diverse global studies.

**Methods:**

This review adhered to the six steps outlined in the guidelines by Askey and O’Malley. Search was conducted across four main databases, including PubMed, JSTOR, Dimension AI, and AJOL, using Medical Subject Headings (MeSH) terms for PubMed and refined for other databases. Additional searches were performed in Google Scholar and university repositories. Reference lists of eligible records were also checked for other relevant articles. Both peer-reviewed and grey literature were included. Search results were screened against predefined inclusion and exclusion criteria, and extraction was done using a data extraction form. Thematic analysis and synthesis were carried out with evidence presented as narrations and summarised in tables.

**Results:**

A total of 15 studies met the inclusion criteria for this review, highlighting socio-cultural factors and interventions affecting TB control. The main socio-cultural factors identified include health literacy, cultural beliefs, socio-economic factors, healthcare accessibility, and cultural practices. Additionally, stigma, financial constraints, reliance on traditional medicine, and inadequate healthcare resources were also identified as contributing factors. Effective interventions focused on community engagement, cultural adaptation, gender sensitivity, and improving accessibility to healthcare.

**Conclusion:**

Socio-cultural factors significantly affect the effectiveness of TB control efforts. Interventions that incorporate community engagement, cultural adaptation, and gender sensitivity are essential for overcoming barriers to care and improving treatment outcomes. Tailoring TB control programmes to address specific cultural contexts and ensuring accessibility and trust in healthcare systems are crucial steps in enhancing global TB control strategies.

## Background

Tuberculosis (TB) remains a leading global health challenge, despite being preventable and curable. The disease disproportionately affects low- and middle-income countries, undermining public health systems and economic productivity (1). Despite decades of intervention, TB persists as one of the top 10 causes of death worldwide, highlighting the need for holistic approaches to address the disease burden (1).

Globally, an estimated 10.6 million people developed TB in 2021, with 1.6 million TB-related deaths recorded (1). Africa bears a disproportionate share of this burden, accounting for 25% of global cases despite having only 17% of the world’s population (2). In Ghana, TB remains a significant public health issue, with an estimated incidence rate of 129 per 100,000 population as of 2023 (3).

TB is a major infectious disease caused by *Mycobacterium tuberculosis*, transmitted primarily through airborne droplets when an infected individual coughs, sneezes, or speaks (1). Several risk factors predispose individuals to TB infection, including overcrowded living environments, undernutrition, HIV co-infection, and compromised immune function (4). Clinically, TB presents with a spectrum of symptoms such as a persistent cough, unintentional weight loss, fever, night sweats, and chest discomfort (5). Delayed diagnosis, often driven by social stigma, misconceptions, and low awareness, exacerbates transmission dynamics and contributes to poorer treatment outcomes (6).

The standard treatment regimen for drug-susceptible TB consists of a six-month course of first-line antibiotics, most notably isoniazid and rifampicin, delivered within the framework of the Directly Observed Therapy Short-Course (DOTS) strategy (7). In contrast, multidrug-resistant TB (MDR-TB) requires longer, more complex regimens involving second-line agents such as bedaquiline and delamanid (8). However, the high costs, limited availability, and toxicity profiles of these medications restrict their use in many low-resource settings (9).

At the global level, the World Health Organisation’s End TB Strategy aims to achieve a 90% reduction in TB-related mortality and an 80% decline in incidence by 2030 compared with 2015 levels (10). This ambitious framework underscores not only biomedical interventions but also the urgent need to tackle social determinants of health, including poverty, limited education, and restricted access to healthcare services. Complementary initiatives such as the Stop TB Partnership and the Global Fund to Fight AIDS, Tuberculosis, and Malaria provide critical support through funding, advocacy, and capacity-building in high-burden regions (11).

Despite these efforts, socio-cultural determinants remain underexplored in the global TB discourse. While existing research acknowledges the importance of factors such as stigma, cultural beliefs, and gender dynamics in shaping health-seeking behaviours and treatment adherence, there has been limited systematic synthesis across diverse geographic and cultural contexts. Furthermore, these determinants are rarely integrated into national policy frameworks or programmatic interventions.

In this regard, the proposed scoping review seeks to fill these gaps by systematically mapping global evidence on socio-cultural influences affecting TB control. Specifically, it will identify recurring themes and regional variations, thereby offering actionable insights for designing culturally sensitive and context-specific TB interventions. This effort directly aligns with Sustainable Development Goal (SDG) 3 of Good Health and Well-being, which emphasises ending the epidemics of AIDS, tuberculosis, malaria, and other communicable diseases by 2030. Ultimately, the review aims to generate evidence that strengthens TB control strategies through the integration of socio-cultural perspectives, ensuring interventions are not only clinically effective but also socially responsive.

## Methods and materials

We conducted this scoping review based on the Arksey and O’Malley framework (12) and reported following the PRISMA extension for Scoping Reviews (PRISMA-ScR) guidelines (13). This review followed six systematic steps which include (1) identifying the research question, (2) identifying relevant studies, (3) selecting studies, (4) charting the data, (5) summarising and synthesising results, and (6) consultation. The protocol for this study has been registered at the Open Science Framework with registration number DOI: https://doi.org/10.17605/OSF.IO/J8VAE.

The following research questions guided this review: (1) What socio-cultural factors influence the prevention, diagnosis, and treatment of tuberculosis across different global contexts? and (2) what interventions are available that address socio-cultural factors?

A systematic literature search was carried out in four major electronic databases: PubMed, JSTOR, Africa Journal Online (AJOL), and Dimension AI. Medical Subject Headings (MeSH) and free-text terms such as “tuberculosis,” “socio-cultural factors,” “stigma,” “health beliefs,” “community perception,” and “gender norms” were used in combination with Boolean operators (AND, OR) to refine the search. Search terms were adapted for each database, and the complete search strategy is presented in Table 1. Grey literature sources, including Google Scholar, WHO Library, and university repositories (Thesis/dissertations), were systematically screened using the same predefined criteria as for peer-reviewed studies. Reference lists of eligible papers were also checked to identify additional records. The search was restricted to articles published between 1990 and April 2025.

**Table 1:** Search strategy in databases used for search.

Database	Search strategy
JSTOR	(tuberculosis OR “pulmonary tuberculosis” OR TB) AND (“socio-cultural” OR sociocultural OR “cultural practices” OR “cultural beliefs” OR stigma OR discrimination OR traditions OR “health-seeking behaviour” OR “religious beliefs”) AND (“tuberculosis control” OR “TB control” OR “treatment adherence” OR “treatment compliance” OR “case detection” OR “DOTS strategy” OR “TB programmes”) AND (global OR worldwide OR international OR “low- and middle-income countries” OR LMICs OR Africa OR Asia OR “high-burden countries”). Search restricted to articles published from 1990 to April 2025.
Africa journals online (AJOL)	tuberculosis AND (“socio-cultural” OR sociocultural OR “cultural beliefs” OR “cultural practices” OR stigma OR “health-seeking behaviour” OR “religious beliefs”) AND (“control” OR “treatment adherence” OR “treatment compliance” OR “case detection” OR “DOTS strategy”). Search restricted to articles published from 1990 to April 2025.
Dimensions AI	TITLE-ABS-KEY((tuberculosis OR “pulmonary tuberculosis” OR TB) AND (“socio-cultural” OR sociocultural OR “cultural beliefs” OR “cultural practices” OR stigma OR discrimination OR traditions OR “health-seeking behaviour” OR “social norms” OR “religious beliefs”) AND (“tuberculosis control” OR “TB control” OR “treatment adherence” OR “treatment compliance” OR “case detection” OR “DOTS strategy” OR “TB programmes”) AND (global OR worldwide OR international OR “low- and middle-income countries” OR LMICs OR Africa OR Asia OR “high-burden countries”))). Search restricted to articles published from 1990 to April 2025.
PubMed	((“Tuberculosis”[MeSH Terms] OR “Pulmonary Tuberculosis”[MeSH Terms] OR tuberculosis[tiab] OR TB[tiab])) AND ((“Culture”[MeSH Terms] OR “Social Stigma”[MeSH Terms] OR “Health Knowledge, Attitudes, Practice”[MeSH Terms] OR “Religion”[MeSH Terms] OR “socio-cultural”[tiab] OR “sociocultural”[tiab] OR “cultural practices”[tiab] OR “cultural beliefs”[tiab] OR stigma[tiab] OR discrimination[tiab] OR traditions[tiab] OR “health-seeking behaviour”[tiab] OR “religious beliefs”[tiab])) AND ((“Tuberculosis, Pulmonary/prevention and control”[MeSH Terms] OR “Patient Compliance”[MeSH Terms] OR “Directly Observed Therapy”[MeSH Terms] OR “case detection”[tiab] OR “treatment adherence”[tiab] OR “treatment compliance”[tiab] OR “DOTS strategy”[tiab] OR “TB programmes”[tiab])) AND (“Developing Countries”[MeSH Terms] OR “Africa”[MeSH Terms] OR “Asia”[MeSH Terms] OR “low- and middle-income countries”[tiab] OR LMICs[tiab] OR global[tiab] OR worldwide[tiab] OR international[tiab] OR “high-burden countries”[tiab])). Search restricted to articles published from 1990 to April 2025.

Search and preliminary screening were conducted with support from a Chartered Librarian. All retrieved citations were imported into Mendeley reference manager for deduplication. Data extraction was performed independently and in duplicate by three reviewers (AA, YS, and FOO) using a pretested data extraction form to ensure consistency and minimise bias. It was piloted on three studies to ensure clarity and reliability before full implementation. Discrepancies between reviewers were discussed and resolved through consensus, with input from a fourth reviewer (PFD) when necessary. Full texts of the shortlisted studies were then reviewed using predefined inclusion and exclusion criteria. Eligible studies included empirical research (qualitative, quantitative, or mixed methods) examining the role of socio-cultural factors in TB-related health outcomes. Articles were excluded if they were opinion pieces, editorials, or focused solely on biomedical or laboratory aspects of TB without exploring cultural or social dimensions.

## Results

### Search results

**Figure 1:**
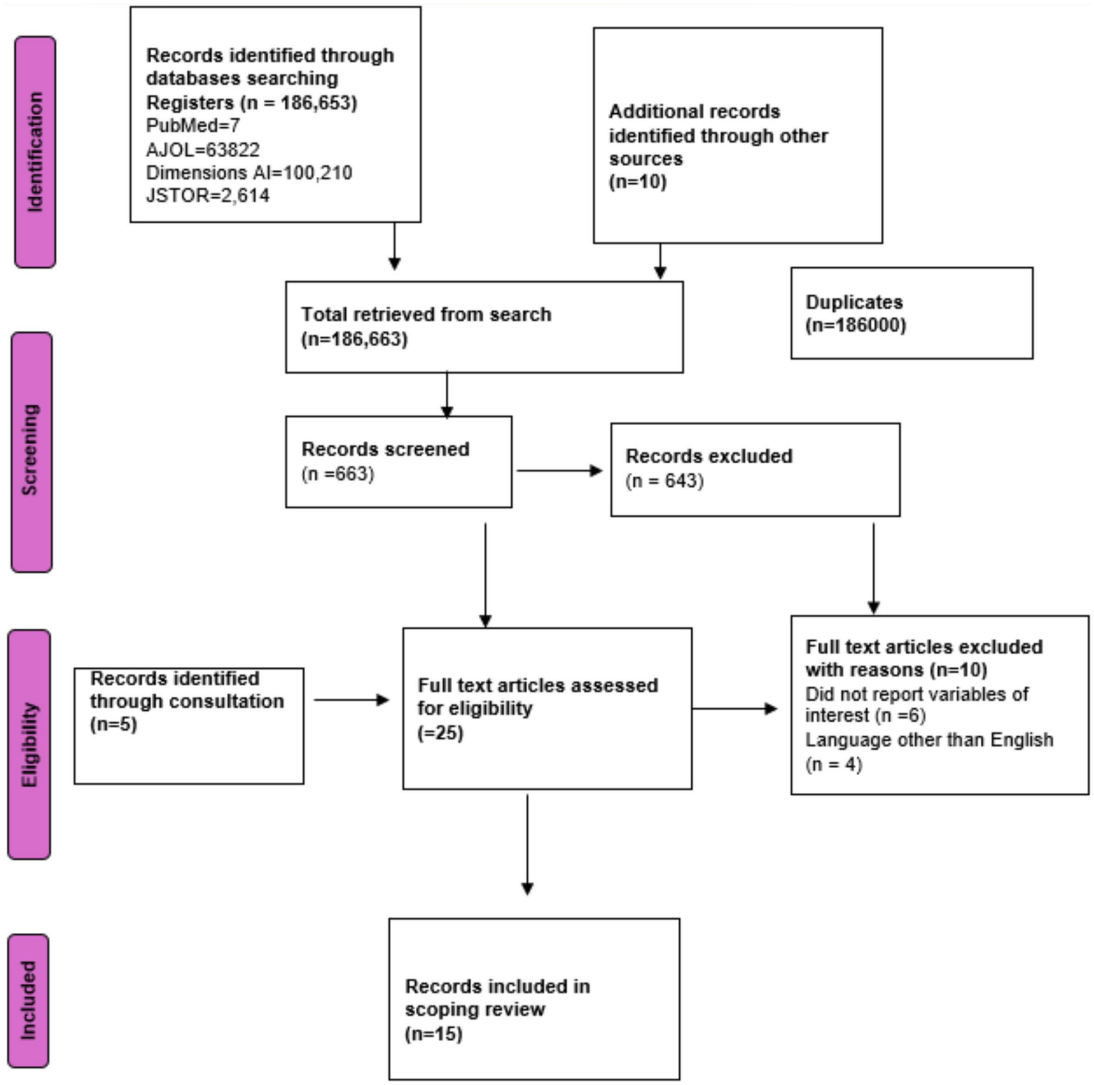
PRISMA flow chart of the search results and screening process.

A total of 186,663 records were identified through database searches, and 10 additional records were retrieved from other sources. Duplicate records, numbering 186,000, were removed prior to screening. After screening 863 titles and abstracts, 643 records were excluded for not meeting the inclusion criteria. This left 220 full-text articles for further assessment. Following eligibility checks, 5 full-text articles were excluded for specified reasons: not reporting variables of interest (*n* = 6), and language other than English (*n* = 4). In addition, 5 records were identified through expert consultation. This brought the cumulative full-text records to 25 for critical screening. Ultimately, 15 records met all eligibility criteria and were included in the scoping review. The PRISMA flowchart is described below in Figure 1.

### Characteristics of included studies

**Figure 2:**
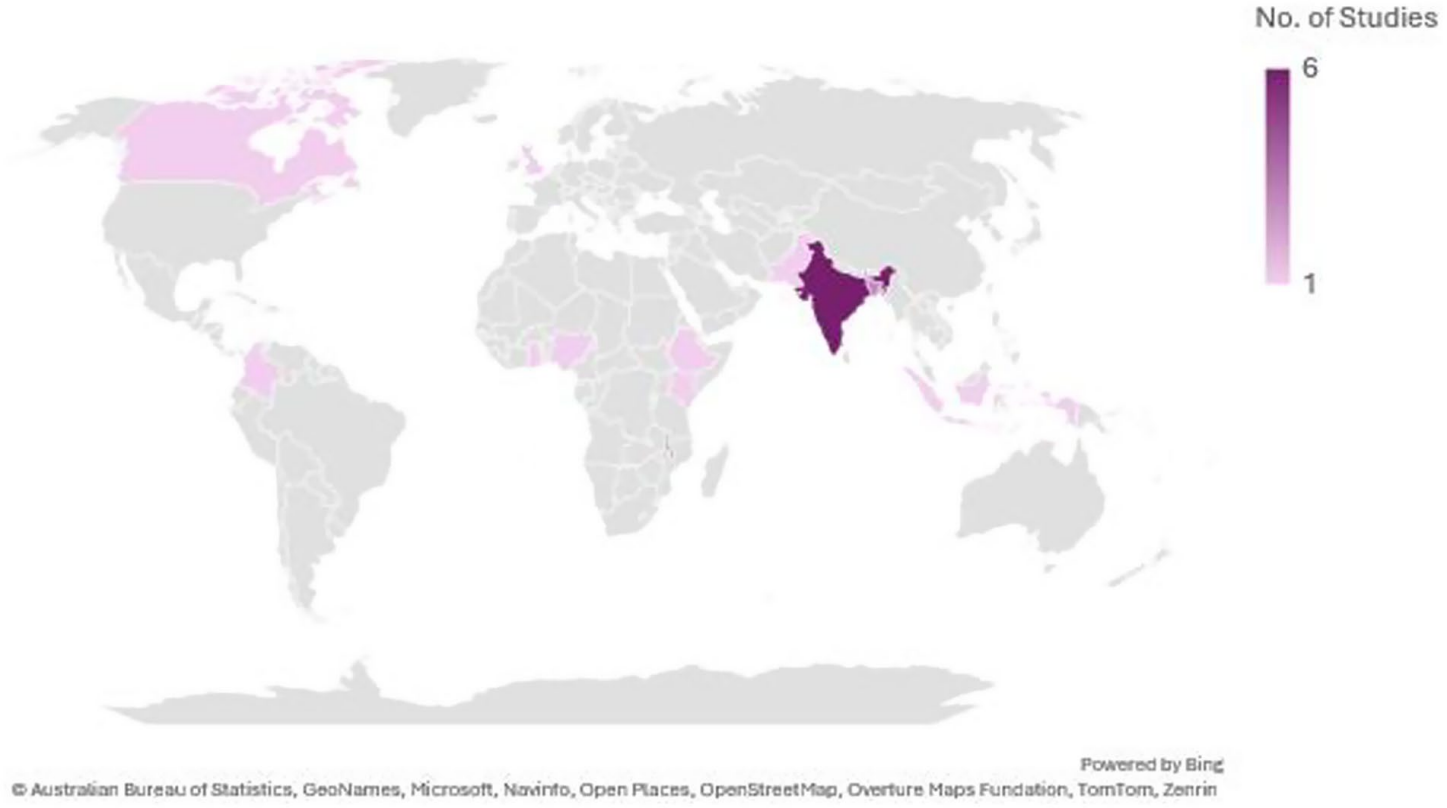
Countries where the included studies were conducted.

**Figure 3:**
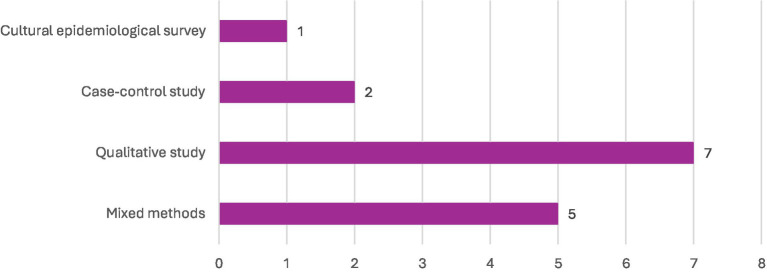
Designs used in the included studies.

**Figure 4:**
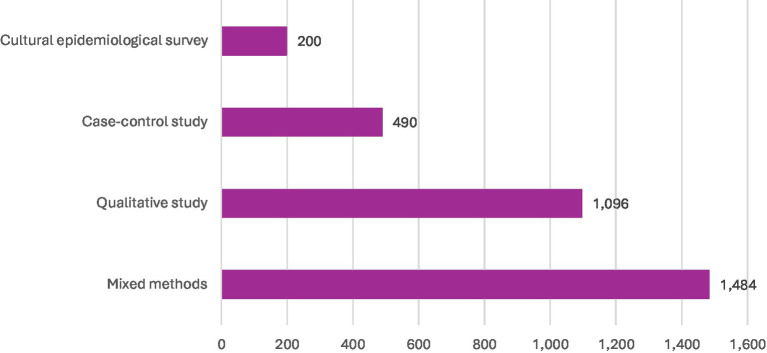
Sample size of included studies according to designs.

**Figure 5:**
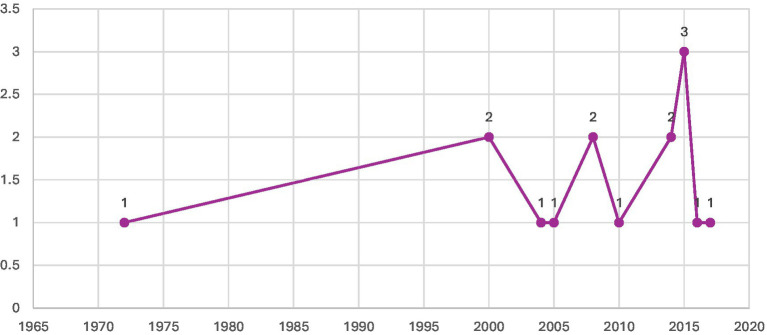
Years of publications of included studies.

Most of the studies included in this review were carried out in India (6), with smaller numbers from Bangladesh (3) and Malawi (2). The remaining studies were single-country contributions from Ethiopia, Canada, Pakistan, Kenya, the United Kingdom, Colombia, Ghana, Nigeria, and Indonesia (see Figure 2 for details). In terms of design, the majority were qualitative studies (7), followed by mixed methods (5), case–control (2), and a single cultural epidemiological survey (see Figure 3). Altogether, the included studies involved 3,270 participants, with the largest sample sizes found in mixed-methods research. Details of sample sizes by study design are shown in Figure 4. With respect to publication years, the earliest study appeared in 1972, with only sporadic publications until the early 2000s. From 2008 onwards, there was a clear increase in output, with notable peaks in 2015 (3 studies) and smaller clusters in 2000 (2) and 2014 (2) (see Figure 5).

### Socio-cultural factors affecting tuberculosis control

Studies reported several socio-cultural factors influencing TB prevention, treatment, and care. Health literacy, cultural beliefs, socioeconomic conditions, healthcare accessibility, and cultural practices collectively shape the risk factors influencing TB knowledge, prevention, and care outcomes. Socio-cultural factors influencing TB control are shaped by diverse dimensions, including health literacy, cultural beliefs, socioeconomic conditions, healthcare accessibility, and cultural practices. Health literacy remains a critical determinant, as limited knowledge of TB symptoms and transmission (14–19), poor awareness of preventive measures (16, 18–20), and barriers to understanding due to communication challenges (21, 22) limit effective disease management. Cultural beliefs equally shape health behaviours, with stigma serving as a major deterrent to disclosure and timely care (15, 21–24), while reliance on traditional healing practices (14, 19, 24, 25) undermines biomedical adherence. Gendered cultural norms further constrain women’s access to diagnosis and treatment (20–23).

Also, socioeconomic factors heighten vulnerability through financial barriers such as inability to afford healthcare and transportation (16, 22), household and environmental exposures including overcrowding and poor ventilation (18, 26), and broader social exclusion which perpetuates marginalisation of the poor and vulnerable (16, 17, 27). Healthcare accessibility challenges reinforce inequalities, with structural barriers to care including long distances and unfavourable health system conditions (14, 16, 20), and language or communication difficulties which compromise patient–provider relationships (15, 17). Finally, cultural practices such as community participation exert mixed effects, sometimes fostering social support but at other times amplifying stigma and exclusion (28) (Table 2).

**Table 2:** Extracted themes and sub-themes from included studies on socio-cultural factors affecting TB control.

Main themes	Sub-themes	Authors
Health literacy	Limited TB knowledge	Gele et al. (14); Gibson et al. (15); Khan et al. (16); Gerrish et al. (17); Karim et al. (18); Taiwo & Adewuyi (19)
	Awareness and prevention	McArthur et al. (20); Khan et al. (16); Karim et al. (18); Taiwo & Adewuyi (19)
	Health literacy barriers	Atre et al. (21); Shiotani & Hennink (22)
Cultural beliefs	Stigma	Atre et al. (21); Gibson et al. (15); Somma et al. (23); Shiotani & Hennink (22); Tabong (24)
	Traditional beliefs and practices	Gele et al. (14); Ndeti (25); Taiwo & Adewuyi (19); Tabong (24)
	Gendered barriers	Atre et al. (21); McArthur et al. (20); Shiotani & Hennink (22); Somma et al. (23)
Socioeconomic factors	Financial constraints	Khan et al. (16); Shiotani & Hennink (22)
	Household and environmental factors	Gosoniu et al. (26); Karim et al. (18)
	Social and economic exclusion	Khan et al. (16); Gerrish et al. (17); Maske et al. (27)
Healthcare accessibility	Barriers to care	Gele et al. (14); McArthur et al. (20); Khan et al. (16)
	Language and communication	Gibson et al. (15); Gerrish et al. (17)
Cultural practices	Social participation	Respati & Sufrie (28)

### Interventions that address socio-cultural factors

**Figure 6:**
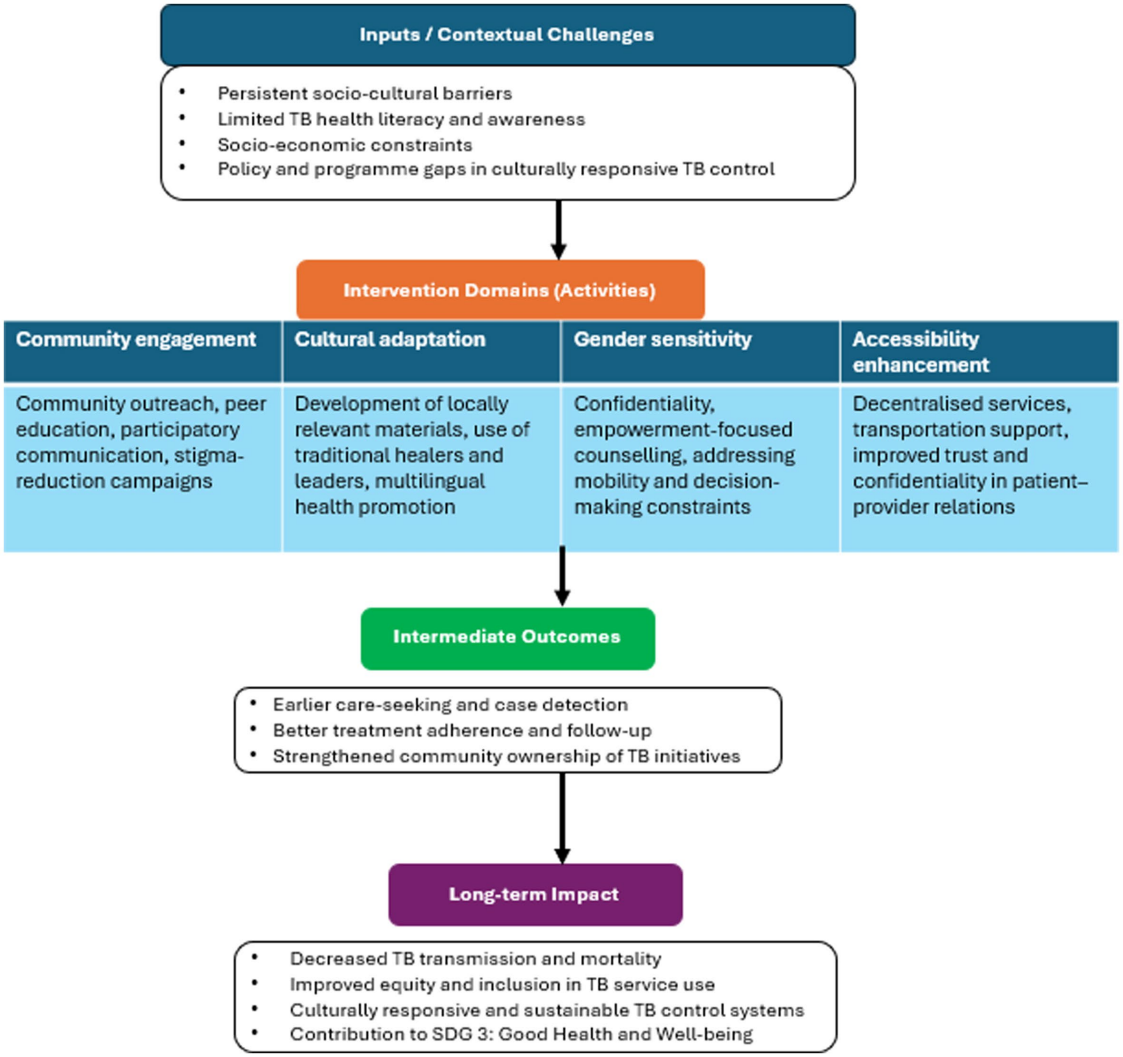
A logic model illustrating socio-cultural interventions.

Studies included in this review reported diverse interventions aimed at improving TB care and addressing socio-cultural barriers across different countries. Community engagement was a central approach, with education and outreach programmes implemented in Ethiopia (14), Canada (15), Pakistan (16), the United Kingdom (17), Bangladesh (18), and Nigeria (19), which enhanced awareness, early diagnosis, and adherence. In India (20), Pakistan (16), Bangladesh (18), and Nigeria (19), these initiatives further promoted participation and shared responsibility for TB prevention. Cultural adaptation strategies, including language modification and culturally sensitive communication, were reported in India (21, 22), while studies from India (21), Canada (15), Malawi and India (23), and Ghana (24) demonstrated that aligning TB messages with local beliefs increased treatment acceptance and trust. Integration of traditional healing systems in Ethiopia (14), Kenya (25), Nigeria (19), and Ghana (24) improved biomedical uptake, whereas gender-sensitive interventions in India (20–23) addressed household decision-making, mobility restrictions, and confidentiality concerns influencing women’s health-seeking behaviour. Accessibility-focused interventions in Pakistan (16) and India (22) included logistical support and transport assistance, and socioeconomic measures in Bangladesh (26) and Bangladesh and India (18) targeted financial barriers and poor living conditions. Enhancing patient–provider trust and reducing treatment default were achieved through interpersonal communication and confidentiality initiatives in Pakistan (16), the United Kingdom (17), and India (27). Lastly, community participation and empowerment strategies in Ethiopia (14), India (20), Pakistan (16), and Indonesia (28) fostered ownership of TB control efforts (Figure 6; Table 3).

**Table 3:** Extracted themes and sub-themes from included studies on interventions to address socio-cultural factors affecting TB control.

Main themes	Sub-Themes	Authors
Community engagement	Community education and outreach	Gele et al. (14); Atre et al. (21); Ndeti (25); Taiwo & Adewuyi (19)
	Community participation and involvement	Respati & Sufrie (28); Tabong (24)
	Sensitization and stigma reduction	Somma et al. (23); Khan et al. (16); Taiwo & Adewuyi (19)
Cultural adaptation	Tailored educational materials	Gibson et al. (15); Gosoniu et al. (26); Maske et al. (27)
	Culturally sensitive communication	Gerrish et al. (17); Shiotani & Hennink (22)
	Integration of traditional approaches	Ndeti (25); Tabong (24)
Gender sensitivity	Addressing gender-specific barriers	McArthur et al. (20); Somma et al. (23)
Improved accessibility	Transportation and logistical support	Shiotani & Hennink (22); Karim et al. (18)
	Confidentiality and trust	Khan et al. (16); Gerrish et al. (17)

## Discussion

### Socio-cultural factors affecting tuberculosis control

The thematic analysis reveals several interconnected challenges that significantly shape TB control, particularly with respect to health literacy, cultural beliefs, socioeconomic constraints, healthcare accessibility, and social practices. Taken together, these themes highlight systemic deficiencies that hinder effective prevention, diagnosis, and treatment. First, inadequate knowledge and poor health literacy about TB are recurring barriers. For instance, Gele et al. (14), Gibson et al. (15), and Khan et al. (16) observed widespread misconceptions regarding TB transmission and heredity, which in turn reinforce stigma and delay care-seeking behaviour. Similarly, McArthur et al. (20) and Taiwo and Adewuyi (19) emphasised that limited awareness of TB’s curability and low levels of symptom disclosure remain common. Consequently, barriers such as a lack of tailored communication strategies and limited community-targeted education, as highlighted by Atre et al. (21) and Shiotani and Hennink (22), leave populations ill-equipped to adopt preventive behaviours.

Beyond health literacy, cultural beliefs and stigma profoundly shape care-seeking decisions. Atre et al. (21) and Somma et al. (23) linked TB-related stigma to gender roles, marital prospects, and its association with HIV/AIDS. In many communities, traditional explanations of TB, such as curses, witchcraft, or divine punishment, undermine trust in biomedical approaches, as reported by Gele et al. (14) and Tabong (24). In addition, gender inequalities further compound these issues. For example, McArthur et al. (20) and Somma et al. (23) revealed that women often face social judgement, confidentiality concerns, and reduced healthcare autonomy, while men’s health tends to be prioritised in household decision-making.

Equally important, socioeconomic and environmental barriers exacerbate TB vulnerability. Khan et al. (16) and Shiotani and Hennink (22) noted that out-of-pocket costs and transportation expenses frequently deter individuals from seeking care. Moreover, poor sanitation, overcrowding, inadequate ventilation, and reliance on biomass fuel, highlighted by Karim et al. (18) and Gosoniu et al. (26), increase transmission risks. In combination with these factors, social and economic exclusion isolates affected individuals, as described by Gerrish et al. (17) and Maske et al. (27), thereby reinforcing dissatisfaction with healthcare and discouraging treatment adherence. Another critical factor relates to healthcare system barriers and patient mistrust. Geographic distance, poor service quality, and long waiting times, documented by Gele et al. (14) and Khan et al. (16), undermine service uptake. Furthermore, mistrust in public health facilities often drives patients to seek private practitioners, even when these services are costlier (20). Similarly, language barriers and cultural misunderstandings, as described by Gibson et al. (15) and Gerrish et al. (17), further limit patient-provider communication, which can reduce treatment adherence and trust in biomedical interventions. Finally, community participation in TB control remains limited. As Respati and Sufrie (28) noted, weak community involvement in health discussions impedes the spread of accurate information and prevents collective ownership of TB prevention efforts. This gap underscores the urgent need for interventions that promote active community engagement, fostering shared responsibility in tackling TB.

### Interventions to address socio-cultural factors affecting TB control

Our review of interventions addressing TB underscores four major interlinked strategies: community engagement, cultural adaptation, gender sensitivity, and improved accessibility. Together, these approaches aim to reduce stigma, enhance equitable healthcare delivery, and overcome systemic barriers to effective TB prevention and treatment. First, community engagement has proven to be central in increasing awareness and fostering shared responsibility. Outreach activities such as mobile health services, community-based education, and participatory communication approaches have been particularly effective in reaching underserved groups (14, 19). Likewise, counselling sessions and peer-led initiatives empower communities to become active partners in TB control, as reported by Respati and Sufrie (28) and Tabong (24). Furthermore, integrating TB education into broader public health campaigns normalises conversations around TB and reduces stigma (23). Second, cultural adaptation enhances both acceptability and effectiveness. Developing multilingual and culturally sensitive materials fosters inclusivity while strengthening providers’ cultural competence, through training or the use of interpreters, and bridges communication gaps (15, 17, 27). Equally, incorporating traditional healers and local belief systems into TB interventions can build trust and improve adherence (24, 25).

Third, gender-sensitive approaches are indispensable. Women, who often face compounded stigma and confidentiality concerns, benefit from interventions that prioritise privacy, promote empowerment, and acknowledge gendered barriers to access (20, 23). Such strategies ensure inclusivity and fairness, enabling more equitable TB outcomes. Finally, addressing accessibility challenges is critical. Strategies such as providing transportation support, reducing structural delays, and decentralising care services directly tackle geographic and financial barriers (18, 22). Moreover, fostering trust through respectful, confidential patient-provider interactions encourages timely treatment-seeking (16, 17).

### Strengths and limitations

This review offers a valuable synthesis of global evidence on socio-cultural factors influencing tuberculosis control, highlighting recurring themes such as stigma, cultural beliefs, gender norms, health literacy, and healthcare accessibility. Its strength lies in the comprehensive and systematic search across multiple databases and grey literature sources, as well as the inclusion of diverse study designs, which collectively provide a broad perspective on contextual barriers and interventions. By mapping evidence from different geographical and cultural settings, the review generates insights that are relevant for shaping culturally sensitive and gender-responsive global TB control strategies. Nonetheless, limitations must be acknowledged. The reliance on English-language publications may have excluded important studies in other languages, and the heterogeneity of included designs poses challenges for comparability. The absence of a formal quality appraisal of included studies and the potential influence of publication bias further limit the certainty of conclusions. Finally, while the global scope enhances generalisability, uneven representation across regions means that some cultural contexts remain underexplored, which may constrain the applicability of findings to underrepresented settings.

### Implications for research, policy, and practice

Future investigations must move beyond descriptive analyses of socio-cultural influences on tuberculosis and focus on robust evaluations of interventions. Implementation research should explore how national and regional TB strategies translate into practice, particularly among marginalised populations. Interdisciplinary research teams comprising public health experts, medical anthropologists, sociologists, behavioural scientists, and health economists are essential to provide deeper insights into stigma, gender inequities, and cultural beliefs. Research councils, universities, and funding bodies such as the Global Fund and WHO’s TDR (Special Programme for Research and Training in Tropical Diseases) are crucial stakeholders in facilitating these studies. Engagement with patient associations and civil society organisations will also ensure that lived experiences inform research priorities.

Policy frameworks must evolve from biomedical dominance to integrated approaches that recognise socio-cultural realities. National Ministries of Health, TB control programmes, and regional health authorities should prioritise stigma reduction, gender-sensitive programming, and culturally adapted health communication. Policymakers must institutionalise partnerships with traditional leaders, faith-based organisations, and local government bodies to enhance community trust and ownership. International stakeholders, including the World Health Organisation (WHO), Stop TB Partnership, and donor agencies such as USAID and the Global Fund, should provide technical and financial support to strengthen health systems and ensure universal access to TB services. Parliamentary health committees and advocacy coalitions also have a role in ensuring accountability, equity, and resource allocation.

At the implementation level, frontline healthcare workers, community health officers, and nurses should be equipped with cultural competence training to provide respectful and inclusive care. Non-governmental organisations (NGOs), community-based organisations (CBOs), and patient support groups are vital in bridging gaps between biomedical care and community expectations. Faith-based groups and traditional healers, when engaged appropriately, can help reduce stigma and reinforce treatment adherence. Development partners and municipal health directorates should support decentralisation through mobile clinics, community diagnostic centres, and logistical support, particularly for rural and disadvantaged areas.

## Conclusion

This scoping review demonstrates that tuberculosis control cannot be divorced from the socio-cultural realities that shape health-seeking behaviours, treatment adherence, and community engagement. Factors such as stigma, gender inequities, traditional beliefs, financial hardship, and mistrust in health systems are not peripheral but central determinants of TB outcomes. The evidence highlights that interventions embedding cultural adaptation, gender sensitivity, and community participation are more likely to foster trust, reduce barriers, and improve adherence. By drawing attention to these dimensions, the review addresses a critical knowledge gap and underscores the necessity for TB strategies that are not only medically sound but also socially responsive. Advancing this agenda requires moving beyond uniform biomedical approaches towards integrated, people-centred frameworks that reflect the lived experiences of affected populations. In doing so, TB control efforts, particularly in African contexts, will be better positioned to accelerate progress towards the Sustainable Development Goal of ending the TB epidemic by 2030.

## Data Availability

The original contributions presented in the study are included in the article/supplementary material, further inquiries can be directed to the corresponding author.
